# Risk factors for insufficient ultrasound-guided supraclavicular brachial plexus block

**DOI:** 10.1186/s40634-023-00611-1

**Published:** 2023-04-20

**Authors:** Shingo Abe, Hiroki Kondo, Yohei Tomiyama, Toshiki Shimada, Masayuki Bun, Kohji Kuriyama

**Affiliations:** 1 Toyonaka Municipal Hospital, 4-14-1 Shibahara, Toyonaka, Osaka 560-8565 Japan; 2 Department of Orthopaedic Surgery, Osaka University Graduate School of Medicine, 2-2 Yamadaoka, Suita, Osaka 565-0871 Japan

**Keywords:** Brachial plexus block, Ultrasound-guided supraclavicular approach, Insufficient block, Risk factors, Additional local anesthesia

## Abstract

**Purpose:**

Ultrasound-guided supraclavicular brachial plexus block (SCBPB) is performed by surgeons for upper limb anesthesia; however, certain patients need additional local anesthesia. This study aimed to identify risk factors for additional local anesthetic injection requirements.

**Methods:**

In total, 269 patients receiving ultrasound-guided SCBPB were enrolled. Patient age, sex, body mass index, anesthetic drug dose, surgeon expertise (hand surgeon or resident), tourniquet time, comorbidities (diabetes mellitus and mental disorders), and preoperative blood pressure representing anxiety were compared between the additional local anesthesia and no additional local anesthesia groups matched for background using propensity scores. Receiver operating characteristic analysis was performed to determine risk factor cut-off values with the highest predictive potential.

**Results:**

Of 269 patients, 41 (15.2%) required additional intraoperative local anesthesia. Among surgical sites, elbow surgery showed the highest prevalence of the need for additional local anesthesia (17/41, 41%). A high body mass index and high systolic blood pressure before surgery were identified as risk factors for additional intraoperative local anesthesia requirement. Furthermore, systolic blood pressure > 170 mmHg (area under the curve, 0.66) predicted the need for intraoperative local anesthesia with 36% sensitivity, 89% specificity, 37.5% positive predictive value, and 88.6% negative predictive value. The median systolic blood pressure was significantly greater in patients requiring additional local anesthesia than in those not requiring it [151 (139–171) mmHg vs. 145 (127–155) mmHg; *P* = 0.026].

**Conclusion:**

Elbow surgery, obesity, and high systolic blood pressure (> 170 mmHg) before surgery are predictive of additional intraoperative local anesthesia requirement.

**Level of Evidence:**

Prognostic Level III

## Introduction

Ultrasound-guided supraclavicular brachial plexus block (SCBPB) is performed routinely by surgeons before upper limb surgery [8, 11] due to the lower risk of hemodynamic instability compared with general anesthesia [13, 15] and the high success rate without further anesthesia requirement [7, 17]. Moreover, surgery with blocks improves postoperative analgesia and avoids discomforts associated with mechanical ventilation. Blocks performed by surgeons may contribute to the reduction of overall medical costs.

We sometimes experience cases with insufficient block effect and require additional local anesthesia during surgery. The incidence of additional local anesthesia due to insufficient block effect ranged from 2 to 38% [7, 14, 17]. However, additional intraoperative local anesthesia increases the risks of adverse effects such as local anesthetic systemic toxicity that would induce central nervous system or cardiovascular system symptoms [3]. The prevalence of local anesthetic systemic toxicity was estimated to be low as 2.5 per 10,000 blockades [3]. Because local anesthetic systemic toxicity was caused by an overdose of local anesthesia, excessive additional local anesthesia injection must be avoided. Regarding factors associated with the block effect, several studies have reported that older age and comorbid diabetes mellitus (DM) prolong block duration [16, 18]. Conversely, other studies have reported that severe anxiety decreases block success [10, 12]. Although a previous prospective study stated that predicting axillary plexus block duration in clinical practice is extremely difficult [4], risk factors for block failure or the need for additional local anesthesia have not been elucidated. The identification of presurgical risk factors for an insufficient block could aid in adjusting the anesthesia dose accordingly or selecting anesthetic combinations to prevent adverse events of local anesthetic systemic toxicity.

We hypothesized that some factors would be related to the insufficient block effect or the need for additional local anesthesia. Thus, this retrospective observational study aimed to identify risk factors for additional local anesthetic injection requirements.

## Methods

This study was approved by the institutional review board and was conducted in accordance with the Strengthening the Reporting of Observational Studies in Epidemiology (STROBE) guidelines. All patients receiving ultrasound-guided SCBPB for upper limb surgery between January 2020 and December 2021 at our institution were included as candidates for the study. The exclusion criteria were as follows: age ≤ 12 years, severe dementia, inability to maintain a resting position during the block procedure and surgery, severe liver or renal dysfunction, and refusal for SCBPB or elected outpatient surgery. All block procedures were performed by one of two hand surgeons who trained for the block procedure for > 2 years or orthopedic residents supervised by one of these hand surgeons. All patients were hospitalized the day before or the day of surgery and were discharged the day after surgery. All patients included in the study provided written informed consent.

### Block procedure

Patients were placed in the supine position with a towel under the back between both scapulas and the head facing the direction opposite to the surgery side. The blood pressure at the contralateral upper limb and vital signs were recorded before the block procedure. Lidocaine 1% and ropivacaine 0.75% (chosen due to fast analgesia onset [2]) were administered under ultrasound guidance using the supraclavicular approach. Briefly, the ultrasound probe (SonoSite S-Nerve, FUJIFILM, WA, USA) was applied along the long axis of the clavicle to view the short axis of the subclavian artery and contiguous brachial plexus. The injection needle (Ultraplex, 22G, 50 mm, B Braun. Melsungen, Germany) was inserted from lateral to medial using the parallel method and advanced to the corner pocket bounded by the subclavian artery, first rib, and brachial plexus. Then, 5–10 mL of the local anesthetic was injected into the corner pocket first, and the injection was repeated 3–5 times around the brachial plexus. In total, 7–10 mL of 1% lidocaine and 14–20 mL of 0.75% ropivacaine were injected, with total doses determined by the surgeon according to the patient’s body weight and age. The entire block procedure required 5–15 min. Additional local anesthesia was administered during surgery when the pain was intolerable, with the administration method and dose decided by the surgeon.

### Evaluation of the block efficacy

Block success (primary outcome) was defined as no further need for anesthesia injection during surgery. To identify factors influencing block efficacy, the following factors were compared between patients requiring or not requiring further intraoperative local anesthesia: total doses of lidocaine and ropivacaine per unit body weight (mg/kg), age, sex, body mass index (BMI), surgeon expertise (hand surgeon or resident), systolic blood pressure before surgery, tourniquet time, DM, and dementia/mental disorders. A study reported that anxiety increases blood pressure [23], so we tried to evaluate preoperative anxiety retrospectively to measure systolic blood pressure before the block procedure.

Sensory and motor blockade were measured every 1–5 min after the administration of the anesthetic to determine the onset time. Sensory blockade was evaluated using the cold test or the pinprick test on skin regions dominated by radial, median, and ulnar nerves. The time between sensation loss and anesthetic administration was measured as the onset time of sensory blockade. Motor blockade was evaluated by the loss of active finger motion, and the time between the complete loss of finger motion and anesthetic administration was measured as the onset time of motor blockade. After surgery, the patients completed a questionnaire to record the time to complete sensory and motor recovery. Patients also graded pain severity using a numerical rating scale (NRS) every 3 h until 12 h after the block procedure. The total sensory and motor block durations were then calculated as the time between onset and recovery. The interval between the termination of anesthetic administration and the start of surgery and the total surgical time were also recorded. The tourniquet inflation time was also measured in patients who required a tourniquet during surgery. The duration of subjective pain block was recorded as the time between the onset of sensory block and the first use of painkillers after surgery. Finally, major complications such as local anesthetic systemic intoxication, pneumothorax, and prolonged paralysis related to nerve injury were recorded.

### Statistical analysis

Logistic regression analysis was used to identify risk factors for additional local anesthesia requirement after SCBPB, with risk expressed as an odds ratio (OR) with a 95% confidence interval (CI). Correction for multiple parameters was not considered because the primary aim was to identify factors that increase the probability of additional local anesthesia requirement during surgery. Differences between the additional local anesthesia and no additional local anesthesia groups were evaluated by the Mann–Whitney U or Chi-squared tests as appropriate. To minimize the influence of group differences in baseline demographic variables, comparisons were also conducted after propensity score matching. The propensity score was calculated by a logistic regression model including age, sex, BMI, operator experience (hand surgeon vs. resident), and comorbid diseases. After propensity score calculation, patients were matched 1:1, yielding 39 pairs.

Receiver operating characteristic (ROC) curves were then constructed for factors significant by logistic regression. The area under the curve (AUC) was calculated, and the optimal cut-off to predict additional local anesthesia requirement was determined using the Youden index. Based on these cut-offs, the sensitivity, specificity, positive predictive value, and negative predictive value for distinguishing the no additional local anesthesia group from the additional local anesthesia group were calculated. A *P* ≤ 0.05 (two-tailed) was considered statistically significant for all tests.

For sample size calculation, we set the alpha, power, effect size, and allocation ratio. The analysis indicated that 40 cases in the additional local anesthesia group were required to achieve a statistical significance level of 0.05, power of 80%, effect size of 0.5, which was referred in Cohen’s d value, and allocation ratio of 4, which was defined by a preliminary study performed in our institution.

## Results

Of the 296 consecutive patients treated at the study site, 27 were excluded according to the predetermined exclusion criteria, and the remaining 269 (115 male and 154 female) patients were included in the analysis. The average patient age was 55 (range, 13–94) years, the average interval from the brachial plexus block procedure to upper arm surgery was 36 (range, 20–69) min, and the average surgery time was 93 (range, 13–323) min. Other clinicodemographic variables are summarized in Table 1. No patients switched to general anesthesia, and sedatives were not administered during surgery. All patients showed no major complications.

**Table 1:** Patients demographics and surgical information (*n* = 269)

	Mean (SD) or n (%)
Age (years)	55.7 (20.7)
Sex	
Male	115 (42.8)
Female	154 (57.2)
BMI (kg/m)	23.1 (4.1)
Lidocaine dose (mg/kg)	1.7 (0.3)
Ropivacaine dose (mg/kg)	2.5 (0.5)
Level of surgeon	
Hand surgeon	177 (65.8)
Residents	92 (34.2)
Comorbidity	
DM	19 (7.1)
Mental disorder	7 (2.6)
Interval from block to surgery (min)	36.1 (7.7)
Surgical time (min)	93.8 (46.2)
Tourniquet inflating time (min)	73.9 (35.6)

*SD* Standard deviation, *BMI* Body mass index, *DM* Diabetes mellitus

**Fig. 1:**
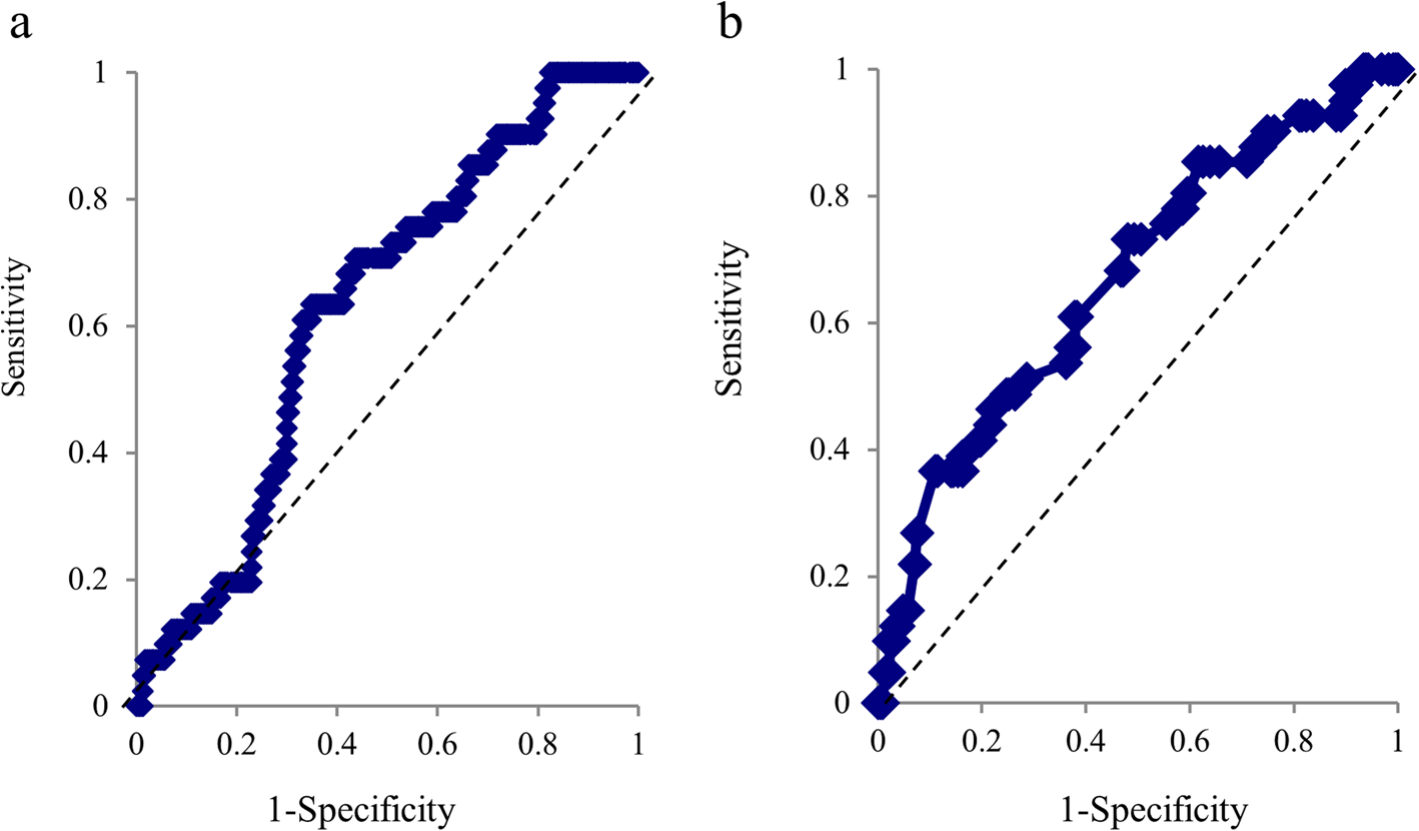
Receiver operating characteristic (ROC) curve for (a) BMI and (b) systolic blood pressure and additional local anesthesia (AUC = 0.62 and 0.66). BMI, body mass index

Of the 269 patients, 41 (15.2%) required additional local anesthesia during surgery. Among surgical sites, elbow surgery had the highest prevalence for additional local anesthesia (17/41 patients, 41%). Table 2 details the surgical sites and percentage of additional local anesthesia. The surgical approaches of 17 patients who underwent elbow surgery and required additional local anesthesia were as follows: 12 patients underwent surgery using the medial approach (in which two cases had the lateral approach), three used the posterior approach, and two used the lateral approach. Local infiltration, intravenous administration, and intravenous regional anesthesia, in addition to a combination of these types, were used for additional local anesthesia. All patients requiring additional local anesthesia were able to undergo surgery. Table 3 shows the details of patients requiring additional local anesthesia. According to the logistic regression analysis, BMI (OR 1.09, 95% CI 1.01–1.19) and systolic blood pressure before surgery (OR 1.03, 95% CI 1.01–1.04) were risk factors for additional local anesthesia during surgery. The results of the logistic regression analysis for other factors are summarized in Table 4. The ROC curve for BMI with a cut-off of 23.8 [AUC = 0.62, sensitivity 63%, specificity 66%, positive predictive value (PPV) 24.8%, negative predictive value (NPV) 90.8%] and systolic blood pressure before surgery with a cut-off of 170 mmHg (AUC = 0.66, sensitivity 36%, specificity 89%, PPV 37.5%, NPV 88.6%) distinguished patients requiring additional local anesthesia from those not requiring it (Fig. 1).

**Table 2:** Surgical procedure and additional anesthesia for each surgical site (*n* = 269)

Surgical site	Number	Number of additional anesthesia (%)
Elbow (*n* = 41)		17 (41.4%)
Bone	12	4
Soft tissue	21	11
Hardware removal	8	2
Forearm (*n* = 14)		1 (7.1%)
Bone	9	1
Soft tissue	3	0
Hardware removal	2	0
Hand & wrist (*n* = 214)		23 (10.7%)
Bone	148	16
Soft tissue	41	3
Hardware removal	25	4

Bone surgery included osteosynthesis of fracture, arthroplasty, and arthrodesis

Soft tissue surgery included ligament, nerve, and infection procedures

**Table 3:** Details of patients requiring additional anesthesia

No	Age	Sex	Surgery site & type	Administration method	Interval from the block to commencement of surgery (min)	Interval from the commencement of surgery to additional anesthesia (min)
1	67	F	Hand, osteosynthesis	LIA	38	2
2	38	M	Hand, tendon, & nerve	LIA	39	0
3	53	F	Hand, hardware removal	LIA	25	0
4	87	F	Hand, osteosynthesis	IVRA	50	0
5	75	M	Hand, tendon transfer	LIA	44	2
6	57	F	Hand, ulnar shortening	IVRA + LIA	50	26
7	62	F	Hand, osteosynthesis	IVRA + LIA	41	44
8	72	F	Hand, osteosynthesis	IVRA	36	42
9	76	F	Hand, osteosynthesis	IVA + LIA	36	0
10	59	F	Hand, osteosynthesis	IVRA + LIA	34	20
11	34	M	Hand, tumor	LIA	39	0
12	21	M	Hand, osteosynthesis	LIA	48	0
13	18	M	Hand, osteosynthesis	LIA	31	0
14	17	M	Hand, osteosynthesis	IVRA	49	0
15	40	M	Hand, osteosynthesis	LIA	33	0
16	70	F	Hand, osteosynthesis	IVA + LIA	36	15
17	63	F	Hand, hardware removal	LIA	28	25
18	58	F	Hand, osteosynthesis	IVRA + LIA	55	0
19	47	F	Hand, osteosynthesis	IVRA + LIA	65	0
20	63	F	Hand, osteosynthesis	LIA	38	0
21	65	M	Hand, bone resection	LIA	41	0
22	57	F	Hand, hardware removal	LIA	30	0
23	73	F	Hand, osteosynthesis	LIA	42	89
24	81	F	Forearm, osteosynthesis	IVRA + LIA	45	13
25	83	F	Elbow, nerve	LIA	33	1
26	15	M	Elbow, hardware removal	LIA	25	1
27	77	F	Elbow, nerve	LIA	34	1
28	77	M	Elbow, nerve	LIA	25	1
29	41	M	Elbow, nerve	LIA	28	1
30	25	M	Elbow, nerve	LIA	37	1
31	38	M	Elbow, ligament	IVRA + IVA + LIA	51	12
32	64	M	Elbow, osteosynthesis	LIA	56	140
33	67	M	Elbow, nerve	LIA	33	0
34	70	F	Elbow, osteosynthesis	LIA	38	1
35	74	M	Elbow, nerve	LIA	30	0
36	41	F	Elbow, bone	LIA	39	6
37	69	M	Elbow, osteosynthesis	IVA + LIA	54	195
38	87	M	Elbow, nerve	LIA	36	0
39	78	F	Elbow, hardware removal	LIA	27	0
40	87	F	Elbow, infection	IVA	37	38
41	73	F	Elbow, osteosynthesis	LIA	38	0

*IVRA* Intravenous regional anesthesia using 0.25% lidocaine 40 mL or 0.5% lidocaine 20 mL

*LIA* Local infiltration anesthesia using 1% lidocaine 3–10 mL

*IVA* Intravenous anesthesia using pentazocine 15 mg

**Table 4:** Logistic regression analysis of risk factors for additional anesthesia requirement

	Regression coefficient	Standard error	Odds ratio	95% CI	*p* value
Age	0.01	0.01	0.99	0.97–1.01	0.271
Sex (Female/Male)	0.03	0.17	1.01	0.54–2.01	0.871
BMI	0.09	1.01	1.09	1.01–1.19	**0.023**
Lidocaine dose	− 0.48	0.47	0.61	0.24–1.56	0.301
Ropivacaine dose	− 0.49	0.32	0.60	0.32–1.15	0.121
Expertise of operator	0.06	0.18	1.14	0.56–2.32	0.713
Systolic blood pressure before surgery	0.03	0.01	1.03	1.01–1.04	** < 0.001**
DM	0.02	0.32	0.95	0.26–3.44	0.945
Mental disorder	0.03	0.54	0.92	0.10–7.89	0.942

*CI* Confidence interval, *BMI* Body mass index, *DM* Diabetes mellitus

*P* values in bold indicate statistical significance (*p* < 0.05)

**Fig. 2:**
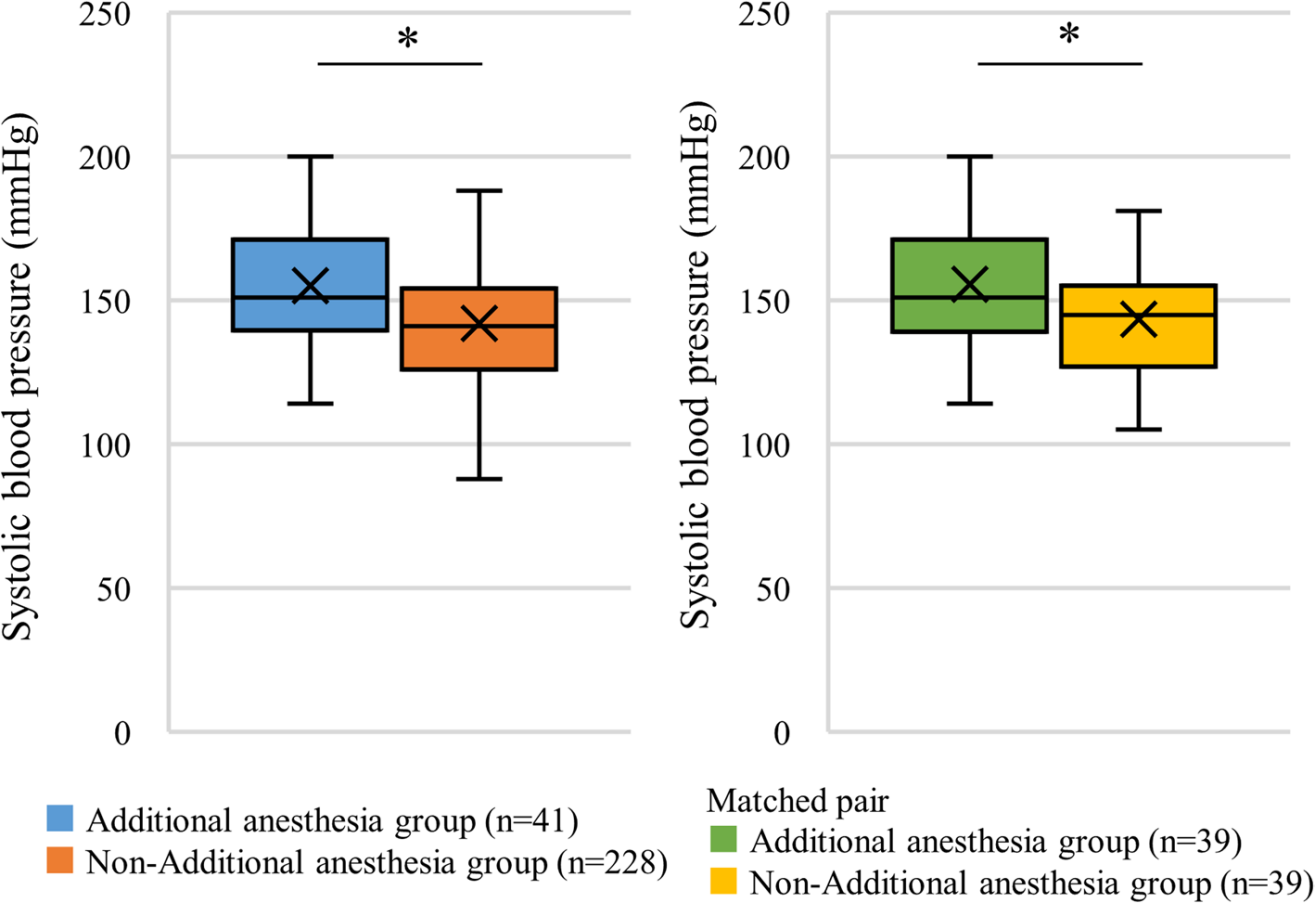
Boxplot comparing systolic blood pressure between 41 patients requiring additional local anesthesia and 228 patients not requiring additional local anesthesia as well as comparing 39 patients requiring additional local anesthesia and 39 propensity-matched patients not requiring additional local anesthesia. Asterisk (*) indicates a significant difference

BMI (median 24.5 vs. 22.5, *P* = 0.009, 1-β = 0.619) and systolic blood pressure (median 151 mmHg vs. 141 mmHg, *P* < 0.001, 1-β = 0.914) were significantly higher in the additional local anesthesia group than in the no additional local anesthesia group. The interval from the block procedure to surgery was significantly longer in the additional anesthesia group than in the no additional anesthesia group. Table 5 presents the differences between the two groups. The groups were then compared after propensity score matching considering age, sex, BMI, operator expertise (hand surgeon or resident), and comorbid diseases, which yielded 39 matched pairs. Within this matched cohort, the median systolic blood pressure was again significantly higher among patients requiring additional local anesthesia (151 [139–171] mmHg vs. 145 [127–155] mmHg; *P* = 0.026, 1-β = 0.704) (Fig. 2). Other factors including the tourniquet time did not show a significant difference between the additional local anesthesia group and the no additional local anesthesia group.

**Table 5:** Comparison of clinical and demographic factors between additional anesthesia and no additional anesthesia groups

	Additional anesthesia group, *n* = 41	No additional anesthesia group, *n* = 228		
	Median [IQR], n (%)	Median [IQR], n (%)	*p* value	1 − β
Age (years)	64 [41–75]	59 [40–72]	0.227	0.191
Sex (male/female)	18/23 (43.9/56.1)	97/131 (42.5/57.5)	0.871	0.039
BMI (kg/m^2^)	24.5 [21.8–25.6]	22.5 [20.0–25.4]	**0.009**	0.619
Lidocaine dose (mg/kg)	1.6 [1.4–1.8]	1.6 [1.4–2.0]	0.325	0.148
Ropivacaine dose (mg/kg)	2.4 [2.1–2.7]	2.5 [2.1–3.0]	0.158	0.291
Expertise of surgeon (Hand surgeon or resident)	28/13 (68.3/31.7)	149/79 (65.3/34.7)	0.713	0.051
Systolic blood pressure before surgery (mmHg)	151 [140–171]	141 [126–154]	** < 0.001**	0.914
DM	3 (7.3)	16 (7.0)	0.945	0.027
Mental disorder	1 (2.4)	6 (2.6)	0.942	0.021
Interval from block to surgery (min)	38 [33–44.5]	36 [31–40]	**0.034**	0.588
Surgical time (min)	83 [57–117.5]	88 [62–121]	0.296	0.162
Tourniquet time (min)	62.5 [43–79.5]	72 [46–101]	0.086	0.460

*BMI* Body mass index, *DM* Diabetes mellitus, *IQR* Interquartile range

1 − β indicated a power

*P* values in bold indicate statistical significance

**Fig. 3:**
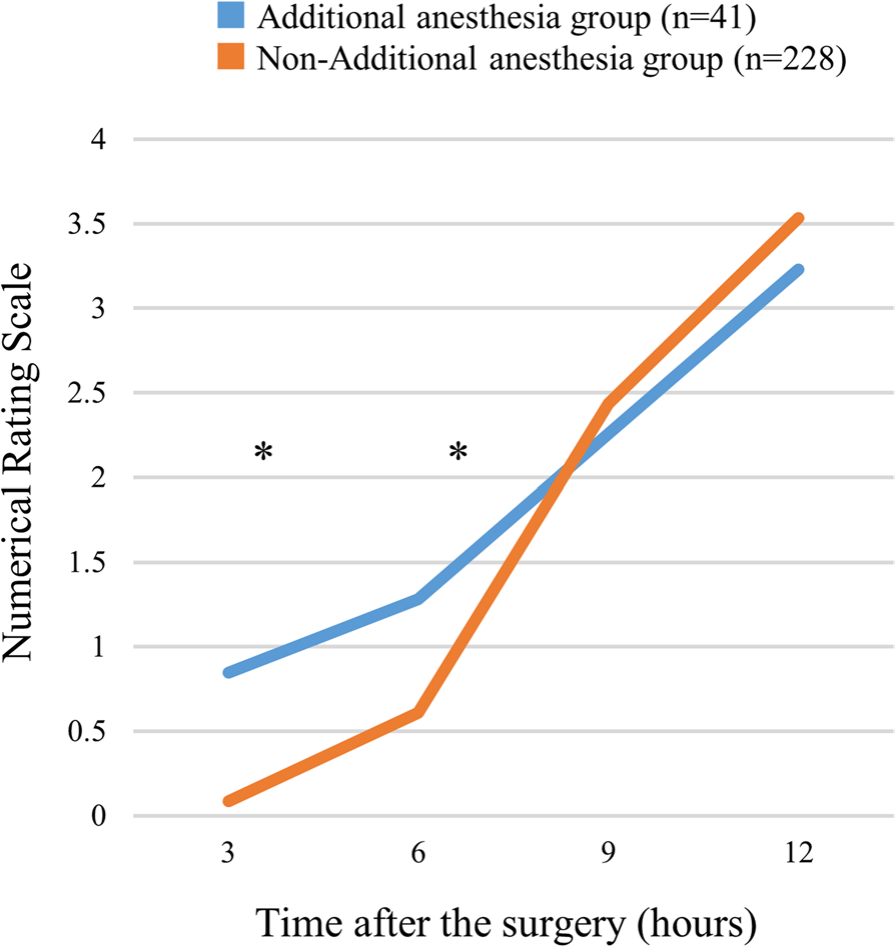
Numerical rating scale for pain completed at 3, 6, 9, and 12 h after surgery by patients requiring additional local anesthesia and patients not requiring additional local anesthesia. Asterisk (*) indicates a significant difference

The average onset times of sensory and motor blockade were 13 ± 13 min and 18 ± 15 min, respectively. The average sensory and motor block durations were 708 ± 292 min and 721 ± 297 min, respectively, and the time from the block until the first postoperative analgesic administration was 615 ± 245 min. The NRS results for pain after surgery are shown in Fig. 3. In general, patients requiring additional local anesthesia reported greater subjective pain levels in the early hours after surgery.

## Discussion

Although identifying factors that influence block duration, particularly factors that reduce the duration or increase the risk of block failure, was difficult [4], we demonstrate that elbow surgery, obesity, and high systolic blood pressure over 170 mmHg before surgery increase the risk for insufficient SCBPB and the requirement for additional local anesthesia during surgery.

The medial side of the humerus, where the medial brachial cutaneous nerve and intercostobrachial nerve dominate, may not be anesthetized by the supraclavicular brachial plexus block [19]; thus, the need for the additional anesthetic to the elbow was reasonable. Actually, our case series of elbow surgery in patients who need additional local anesthesia is mainly related to the medial approach of the elbow. Obesity was also identified as a risk for additional local anesthesia. A previous study reported a higher block failure rate in patients with obesity [20]. Given that the brachial plexus tends to be located deeper, due to fat or a shorter neck in patients with obesity, puncturing the block needle around the nerve is technically difficult. This may cause insufficient block efficacy. Moreover, ultrasound-guided SCBPB would cause ulnar sparing in approximately 18%–30% [1, 5] and some of the patients who required additional local anesthesia in our cohorts might experience ulnar sparing. A study reported that anxiety increases systolic blood pressure, and anxiety is one of the reasons of the insufficient block effect [10, 12, 23]. Therefore, high systolic blood pressure due to severe anxiety would be a risk factor for insufficient block effect. The cut-off value has not been reported, and we suggest systolic blood pressure over 170 mmHg as an independent risk factor for additional local anesthesia requirement. The ROC analysis revealed that systolic blood pressure could distinguish patients requiring additional analgesia with high specificity (89%) albeit with low PPV (37.5%) because of the low prevalence of additional local anesthesia requirement (15%). Post hoc power analysis further indicated that systolic blood pressure had a high power (0.914 for the no additional local anesthesia group and 0.704 for the matched-pair no additional local anesthesia group) and a relatively low type 2 error. Although DM and older age were reported to prolong block duration [16, 18], DM and older age were not found to be significant risk factors of additional local anesthesia in our cohorts. The prevalence of DM is relatively low in our cohorts; therefore, it did not indicate a significant difference.

The proportion of patients requiring additional local anesthesia during surgery varies markedly across study cohorts. Regarding additional anesthesia prevalence, Perlas et al. [17] reported the need for additional local anesthesia in 2.8% (13 of 460) of patients undergoing upper limb surgery and for general anesthesia in 2.6% (12 of 460). Obata et al. [14] reported that 38.6% (39 of 101) of patients with distal radius fractures required additional intraoperative local anesthesia, and 5.9% (6 of 101) were converted to general anesthesia. Furthermore, Gamo et al. [7] reported that 25.4% (51 of 202) of patients undergoing upper limb surgery required additional anesthesia, and 0.5% (one of 202) were converted to general anesthesia. In our patient series, 15% (41 of 269) required additional local anesthesia, within the range of these previous reports. We also assume that block efficacy and pain could be accurately assessed because no cases were switched to general anesthesia and sedation drugs in our cohort.

The time interval from block to surgery was longer in the additional local anesthesia group than in the no additional local anesthesia group. It may be because the surgeon would delay the skin incision if the block effect is insufficient for the patient to tolerate the pain of the skin incision. Because this is a retrospective study, the time between the block procedure and the start of surgery was not prespecified. Of 41 patients, 29 required additional local anesthesia at the skin incision; thus, we assumed that the block was delayed in this subgroup. Moreover, 12 of these 41 patients required anesthesia > 10 min after the skin incision and were able to move their fingers during the administration of additional local anesthesia, suggesting that the block wore off prematurely.

The block duration is dependent on the pharmacokinetics of the analgesic agents used. Ropivacaine is metabolized in the liver and excreted in the kidney [9]. The peak plasma concentration was observed approximately 1 h after injection, and the half-decay period was approximately 6 h following axillary block [22]. The average time to anesthesia onset was approximately 15 min, and the block duration was approximately 10–12 h in this patient series, within ranges reported previously for ropivacaine (onset range 12–45 min for 2.7 ± 0.2 mg/kg; mean duration 13.5 h [4.8–25.4 h] for 3.6 ± 0.8 mg/kg; 6 h for 1% prilocaine 20 mL plus 0.75% ropivacaine 20 mL, and 12 h for 0.75% ropivacaine 40 mL) [4, 6, 21]. Given that the proportion of patients requiring additional local anesthesia after SCBPB was also within the mid-range of previous studies, this patient cohort is likely a strongly representative sample.

This study has several limitations. First, relatively few patients required additional local anesthesia, limiting the statistical power for the identification of other risk factors. Second, the primary outcome was assessed by the need for additional local anesthesia during surgery due to pain intolerance, which can vary markedly among individuals and was not standardized. However, the presence of finger motion in some patients of the additional local anesthesia group suggests true anesthesia insufficiency. Third, the block procedure was performed by several surgeons (hand surgeons/residents), and body characteristics such as neck length and brachial plexus depth were not standardized, introducing additional sources of variability. However, the hand surgeons trained for the block procedure for > 2 years, so we believed that their skills were guaranteed. Finally, the groups included all kinds of surgery: bone surgery, soft tissue surgery, and hardware removal. This heterogeneity might affect the results. However, additional anesthesia was conducted in all kinds of surgery, as shown in Table 2. The most influential factors include surgical site and patient factors such as obesity and anxiety.

## Conclusions

In summary, we identified elbow surgery, obesity, and systolic blood pressure over 170 mmHg before surgery as risk factors for additional local anesthesia requirement during surgery. The block failure associated with high blood pressure would be caused by preoperative anxiety.

## Data Availability

The datasets generated and analyzed during the current study are not publicly available due IRB decision but are available from the corresponding author on reasonable request.

## Abbreviations

AUC: Area under the curve
BMI: Body mass index
CI: Confidence interval
DM: Diabetes mellitus
NRS: Numerical rating scale
OR: Odds ratio
ROC: Receiver operating characteristic
